# Annual and seasonal spatial models for nitrogen oxides in Tehran, Iran

**DOI:** 10.1038/srep32970

**Published:** 2016-09-13

**Authors:** Heresh Amini, Seyed-Mahmood Taghavi-Shahri, Sarah B. Henderson, Vahid Hosseini, Hossein Hassankhany, Maryam Naderi, Solmaz Ahadi, Christian Schindler, Nino Künzli, Masud Yunesian

**Affiliations:** 1 Department of Epidemiology and Public Health, Swiss Tropical and Public Health Institute, Basel, Switzerland; 2 University of Basel, Basel, Switzerland; 3 Research Center for Environmental Pollutants, Qom University of Medical Sciences, Qom, Iran; 4 Department of Epidemiology and Biostatistics, School of Public Health, Isfahan University of Medical Sciences, Isfahan, Iran; 5 Environmental Health Services, British Columbia Centre for Disease Control, Vancouver, Canada; 6 School of Population and Public Health, University of British Columbia, Vancouver, Canada; 7 Mechanical Engineering Department, Sharif University of Technology, Tehran, Iran; 8 Tehran Air Quality Control Co., Tehran Municipality, Tehran, Iran; 9 Center for Air Pollution Research (CAPR), Institute for Environmental Research (IER), Tehran University of Medical Sciences, Tehran, Iran

## Abstract

Very few land use regression (LUR) models have been developed for megacities in low- and middle-income countries, but such models are needed to facilitate epidemiologic research on air pollution. We developed annual and seasonal LUR models for ambient oxides of nitrogen (NO, NO_2_, and NO_X_) in the Middle Eastern city of Tehran, Iran, using 2010 data from 23 fixed monitoring stations. A novel systematic algorithm was developed for spatial modeling. The R^2^ values for the LUR models ranged from 0.69 to 0.78 for NO, 0.64 to 0.75 for NO_2_, and 0.61 to 0.79 for NOx. The most predictive variables were: distance to the traffic access control zone; distance to primary schools; green space; official areas; bridges; and slope. The annual average concentrations of all pollutants were high, approaching those reported for megacities in Asia. At 1000 randomly-selected locations the correlations between cooler and warmer season estimates were 0.64 for NO, 0.58 for NO_X_, and 0.30 for NO_2_. Seasonal differences in spatial patterns of pollution are likely driven by differences in source contributions and meteorology. These models provide a basis for understanding long-term exposures and chronic health effects of air pollution in Tehran, where such research has been limited.

Air pollution is a complex mixture of gases and particles, and it has been associated with a wide range of health outcomes^1^,^2^. The latest estimates from the Global Burden of Disease (GBD) Study indicated that approximately 87% of the global population is exposed to ambient concentrations of fine particulate matter (PM_2.5_) that do not meet the guideline values set by the World Health Organization (WHO)^3^,^4^. This estimate is even higher when restricted to the populations of low- and middle-income countries (LMICs). In addition, air pollution was one of six modifiable risk factors associated with more than 5% of the GBD, as measured by disability-adjusted life years lost (DALYs)^5^. This burden is also reflected in Iran^6^,^7^, where the latest estimates suggest that approximately 7% of total DALYs are attributable to air pollution, which is ten times greater than the DALYs attributable to HIV/AIDS and tuberculosis combined^8^. Even so, the burden of air pollution might be substantially underestimated because (1) most of the exposure-response estimates are from high-income countries, and (2) the burden might not be fully captured by PM_2.5_ and ozone, which were the only indicators used in the GBD analyses. Furthermore, emerging evidence suggests that air pollution is associated with many chronic diseases not yet included in the GBD assessment, such as acceleration of atherosclerosis^2^, high blood pressure^9^,^10^, diabetes^11^,^12^, metabolic syndrome^13^, and possibly with neurodegenerative diseases such as multiple sclerosis^14^, vascular dementia and Alzheimer’s disease^15^,^16^.

The scientific community has consistently stated that lack of epidemiologic evidence from LMICs limits the generalizability of current air pollution findings^4^. One pillar of air pollution epidemiology is high quality exposure estimates^17^, but quantification of exposures at the individual level has been especially challenging in LMICs^18^,^19^,^20^. In light of the long-term health effects associated with reduced air quality, methods that estimate the spatial distribution of air pollutants are particularly useful. Land use regression (LUR) is a widely applied, state-of-the-science method used to map spatial variability in ambient air pollutants. Generally speaking, LUR uses local land use information characterized in geographic information systems (GIS) to estimate concentrations of air pollutants at any location within a city^21^. The land use variables can represent a broad range of characteristics in the area surrounding the locations, such as the type of land use, elevation, population density, point sources, and vehicle traffic^22^. Valid LUR models offer the opportunity to estimate air pollution concentrations at locations where no measurement data are available.

Two important considerations in LUR modeling are (1) the number of monitoring sites and (2) the locations of those sites within the study area^23^. Some LUR models are based on data from a small number sites (for example, 17 sites in Houston metropolitan area)^24^, whereas others use large numbers of local, national, or multi-national measurement locations (for example, 562 sites across 12 Spanish cities in Girona Province^25^ or 2400 sites across Europe)^26^. Previous work suggests that LUR models should be constructed using measurements from at least 80 locations^27^ identified by some algorithm to optimize their spatial variability^28^. However, several studies have used secondary data from existing regulatory monitoring networks, which typically have fewer locations^24^,^29^,^30^,^31^,^32^ with less spatial variability. In addition to these considerations, Basagaña *et al*. (2012) suggested that LUR analyses should use a restricted set of predictor variables, especially when the number of monitoring sites is small^27^. However, no LUR study to date has introduced a systematic approach to restricting the variable set.

Nitrogen oxides (NOx) are a group of highly reactive gasses that contain different numbers of nitrogen and oxygen atoms, including nitrogen oxide (NO), nitrogen dioxide (NO_2_), and nitrous oxide (N_2_O). However, NOx is frequently considered to be the sum of NO and NO_2_ in atmospheric sciences^33^. Fossil fuel combustion produces NO^34^ as a primary pollutant. This free radical rapidly oxidizes in the atmosphere, scavenges tropospheric ozone, and converts to secondary NO_2_^35^. Both mobile and point sources contribute to NO in Tehran. Iran benefits from large natural gas reserves^36^ that are used for most commercial processes and residential heating.

To date, LUR has been applied to model NO_2_^21^,^22^,^30^,^37^,^38^,^39^ in many high-income countries and in some LMICs^40^,^41^. However, LUR models for NO and NOx are rare, especially in LMICs^42^. We previously reported LUR models for particulate matter (PM_10_) and sulfur dioxide (SO_2_) in Tehran, where the entire population was located in areas exceeding the WHO guidelines for both pollutants^40^. The results also suggested the potential for seasonal differences in the spatial patterns of more primary pollutants. Here we develop annual and seasonal models for NO, NO_2_ and NOx using data from the regulatory monitoring network.

## Results

### Air quality data

**Figure 1:**
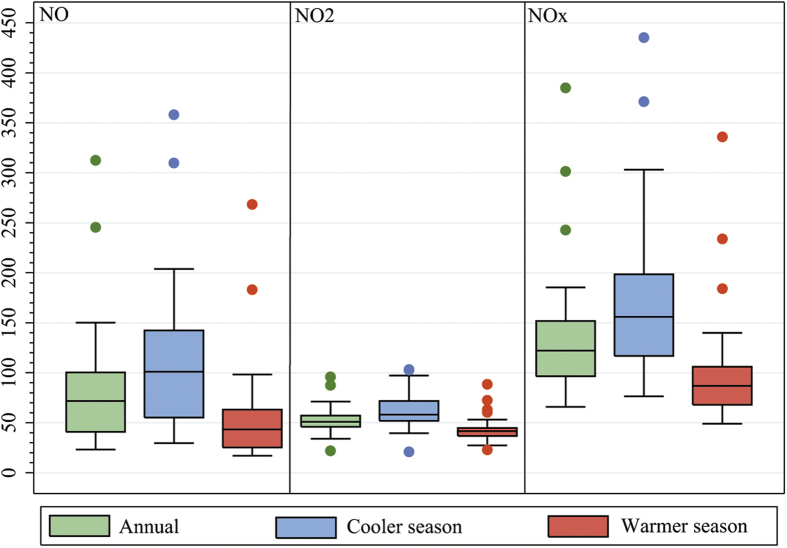
Distribution of pollutant concentrations (ppb) over the 23 monitoring stations in Tehran, Iran, 2010. The figure is generated using STATA 13 (STATA Corp., TX, USA, http://www.stata.com/).

None of the pollutants were normally distributed (*p* < 0.001). The annual median concentrations (interquartile range, or IQR) were 71.7 (59.3) ppb for NO, 50.9 (11.1) ppb for NO_2_, and 122.3 (55.1) ppb for NO_X_ across the 23 monitoring stations. The cooler season medians (IQR) were 100.9 (87), 58.1 (19.7), and 155.9 (81.6) ppb, respectively, and the warmer season values were 43.4 (37.7), 41.7 (7.9), and 87.0 (38.0) ppb, respectively (Fig. 1). The correlation between the annual, cooler season, and warmer season concentrations ranged from 0.94 to 0.99 for NO, from 0.61 to 0.92 for NO_2_, and from 0.90 to 0.99 for NO_X_ across the 23 monitoring stations. The between-pollutant correlations ranged from 0.25 to 0.46 for NO and NO_2_, from 0.85 to 0.95 for NO and NO_X_, and from 0.38 to 0.72 for NO_2_ and NO_X_ (Table S2, supplemental information).

### Final LUR models

Of the 210 potentially predictive variables (PPVs) we generated, 21 (10%) were significantly predictive in one or more of the LUR models. The R^2^ values for the final annual mean models were 0.78, 0.69, and 0.71 for NO, NO_2_ and NO_X_, respectively. They ranged from 0.69 to 0.79 for the cooler season models and 0.61 to 0.72 for the warmer season models (Table 1). Some of the variables appeared in multiple models. These included: (1) distance to the traffic access control zone; (2) distance to sensitive land use areas; (3) the natural logarithm of distance to the nearest primary school; (4) the natural logarithm of distance to the nearest hazardous facility; (5) slope; (6) the presence of bridges, and (7) areas of green, official/commercial, and other land uses (Table 1, and Tables S3–S11, supplemental information). The Moran’s I results were −0.07, −0.12, and −0.04 for residuals of the annual, cooler season, and warmer season NO models. All p-values were greater than 0.23. The values were similar for the NO_2_ and NOx models (not shown), with a minimum p-value of 0.06.

### Model stability

The R^2^ values for the leave-one-out cross validations ranged from 0.53 to 0.66 for the NO models, 0.51 to 0.58 for NO_2_ models, and from 0.42 to 0.63 for the NO_X_ models (Table 1, and Tables S3–S11, Supplemental information). A final leave-one-out cross-validation (LOOCV) check was done for the coefficient of each predictive variable in the final regression models. The minimum and maximum of the LOOCV coefficients had the same direction of effect for all variables in all models. All the coefficients of variation ranged from 7% to 11%.

### Regression maps

The limits of prediction for the annual, cooler season, and warmer season NO models were 16.4, 21.0, and 12.0 ppb, respectively. For the NO_2_ models they were 15.6, 14.9, and 16.3 ppb, respectively, and for the NOx models they were 46.6, 54.0, and 34.6 ppb, respectively. Overall, out of 24,505,474 grid cells in the modeling domain, a range of 0.2% to 16.0% of cells were increased to the limit of prediction and 0.0% to 5.3% of cells were truncated to 120% of the maximum observed concentrations (Table 2).

**Figure 2:**
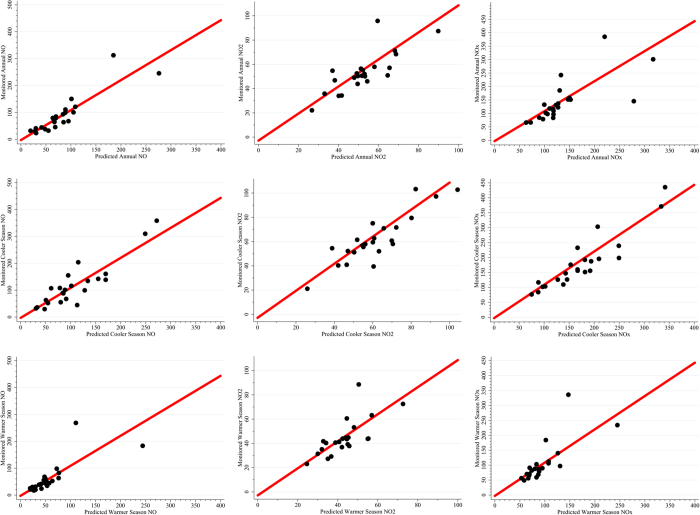
Observed versus predicted concentrations (ppb) for annual, cooler and warmer seasons of NO, NO_2_, and NO_X_ in Tehran, Iran. The red line is the 1:1 linear prediction. The figures are generated using STATA 13 (STATA Corp., TX, USA, http://www.stata.com/).

**Figure 3:**
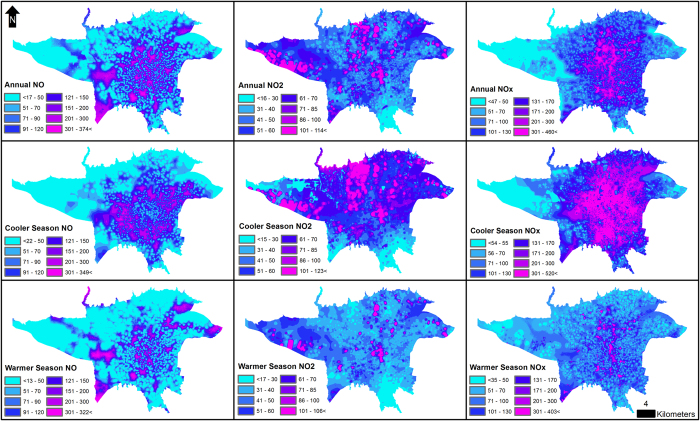
Estimated annual, cooler and warmer seasons NO, NO_2_ and NO_X_ concentrations (ppb) from the final land use regression models in Tehran, Iran, 2010. The *prediction resolution* is 5 × 5 meters. The figure is generated using ESRI’s ArcGIS 10.2.1 for Desktop (ESRI, Redlands, CA, USA, http://www.esri.com/).

Agreement between the measured and predicted pollutant concentrations was relatively good (Fig. 2). The maps showed clear hotspots for the NO concentrations across the city. These were well-characterized by distance to the traffic access control zone, the natural logarithm of distance to the nearest primary school, surrounding areas of green land use, and slope. The NO_2_ concentrations were more dispersed and homogeneous throughout the city. The NO_X_ maps were similar to the NO maps, and the hotspots were driven by similar variables, though they also reflected distance to the nearest hazardous facility and the presence of bridges (Fig. 3).

**Figure 4:**
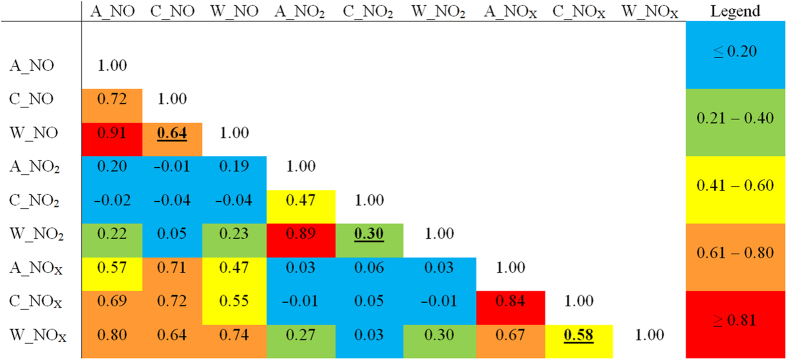
The Spearman correlation coefficients between the annual (**A**), cooler season (**C**), and warmer season (**W**) predicted concentrations across 1000 random locations for NO, NO_2_, and NOx in 2010, Tehran, Iran. The seasonal comparisons (**C** vs **W**) are bold underlined.

The correlations between the predicted annual, cooler season, and warmer season concentrations at 1000 randomly-selected sites were weak to moderate. Values for the warmer and cooler season estimates were 0.64 for NO, 0.58 for NOx, and 0.30 for NO_2_ (Fig. 4).

## Materials and Methods

### Study area

**Figure 5:**
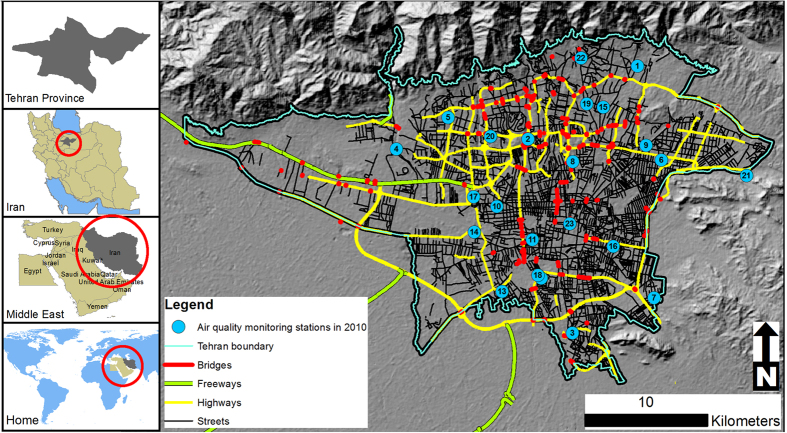
The study area of Tehran, Iran showing locations of 23 air quality monitoring stations in 2010. The figure is generated using ESRI’s ArcGIS 10.2.1 for Desktop (ESRI, Redlands, CA, USA, http://www.esri.com/).

The megacity of Tehran is the capital of Iran. It covers an area of 613 km^2^, with the Alborz Mountains in north and desert in south. The populated areas within the city range from 1,000 to 1,800 meters above sea level (Fig. 5). The annual mean daily temperature is 18.5 °C, with highs of 43 °C in July and lows of −15 °C in January. The average annual precipitation is 220 millimeters (mm), with the maximum in March (39 mm) and the minimum in September (1 mm). The weather is typically sunny, with an annual average of 2800 h of bright sunshine and a mean cloud cover of 30%. The prevailing winds blow from west and north (Figure S1, Supplemental information). Tehran is the most populous city in Iran, and the third largest city in the Middle East. There are approximately 9 million urban residents, with a daytime population of more than 10 million due to diurnal migration from the surrounding areas^40^,^43^.

### Air quality data

Hourly NO, NO_2_ and NOx concentrations for the 2010 calendar year were obtained from 23 air quality monitoring stations administered by two government agencies (Fig. 5). Of the stations, 16 belonged to the Air Quality Control Company (AQCC), and 7 to the Department of Environment (DOE). Both the AQCC and DOE monitoring stations used chemiluminescence analyzers (Model AC 32 M of Environment SA, France; APNA-370 of Horiba, Japan; and EC 9841 of Ecotech, Australia) to measure nitrogen oxides. They follow quality assurance/quality control (QA/QC) procedures that, under ideal circumstances, ensure the instruments are checked and calibrated every two weeks. However, calibration gases can be challenging to obtain in Tehran.

A complete annual dataset would contain 8760 measurements (24 hours/day × 365 days in 2010) for each pollutant at each monitoring site. However, 28.1%, 27.7%, and 27.6% of the NO, NO_2_ and NO_X_ values were missing, respectively (Figure S2, supplemental information). As in our previous work^40^, the Amelia program was used for imputation of the missing data (Page S5, supplemental information)^44^. The program uses a new expectation-maximization algorithm with bootstrapping to impute missing values and return a complete dataset. We provided the program with all available hourly concentrations from the different stations, along with the month, day, and hours of measurement. In order to evaluate the consistency and reliability of the missing data estimates we ran the Amelia program 10 times for each pollutant to impute hourly missing values, and calculated the resulting 10 annual averages for each monitoring station. The mean of the 10 imputation-filled datasets was calculated for NO, NO_2_ and NO_X_ from January 1^st^, 2010 through January 1^st^, 2011 for all monitors, and these values were used as the LUR response variables.

We also divided the year into warmer and cooler seasons based on our previous work^40^ and because Chen *et al*. (2010) reported different LUR predictor variables and spatial patterns in Tianjin, China during the heating and non-heating seasons. The same study also found that the predictive variables and the R^2^ values for the LUR models differed by season^41^. The warmer and cooler seasons were defined as April through September and October through March, respectively. These months were selected based on WHO guidelines for countries in the Northern hemisphere, and on the highest and lowest mean daily temperatures at Mehrabad International Airport in Tehran^40^.

### Spatial predictors

We generated 210 PPVs in six classes and 73 sub-classes (Table 3). The six classes were *Traffic Surrogates, Land Use, Distance Variables, Population Density, Product Variables, and Geographic Location*. The *Traffic Surrogates* class described the vehicular network in buffers around the pollution monitoring stations. The *Land Use* class described ten land use types within buffers around the stations. The *Distance Variables* class measured the Euclidian distance (and natural logarithm of the distance) from each station to all of the *Traffic Surrogate* and *Land Use* types, and to other features. The natural logarithms of the distances were used based on studies that have reported exponential decay in air pollutant concentrations with increasing distance from pollution sources^45^,^46^,^47^,^48^. The *Population Density* was calculated for the total population and for the population excluding unemployed people and children less than five years of age. The *Product Variables* class included the ratio of variables in the *Traffic Surrogates* class to the variables in the *Distance Variables* class. Finally, the *Geographic Location* class included the elevation of each monitoring site, obtained from a digital elevation model (DEM) of Tehran in meters above sea level, and a slope (gradient) variable that was created in GIS based on the DEM. The potential geospatial variables were selected based on previous studies and available information in Tehran. The raw GIS inputs were all in vector format, originating from the Japan International Cooperation Agency (JICA) and the Centre for Earthquake and Environmental Studies of Tehran (CEST)^49^. The final PPVs were all in raster format with a resolution of 5 × 5 meters, and their values in the grid cells underlying the monitoring stations were used for the regression analyses. All spatial analyses and figures were generated using ESRI’s ArcGIS 10.2.1 for Desktop (http://www.esri.com/).

### Model development and diagnostics

The model building algorithm was based on one we developed for a previous study^40^. However, we further refined the algorithm to account for non-normality of the response variable, which can violate the assumptions of linear regression modeling. We also used transformation to normalize the relationships between the response variables and the PPVs, and we restricted the number of variables in the final model to the root of the number of observations. The key steps of the updated stepwise algorithm are:

1. Take the log transformation of the response variable.
2. Check for normality using the Shapiro-Wilk test^50^.
3. Apply a power transformation if not normally distributed.
4. Linearize the relationships between the transformed variables and the PPVs using log and power transformations on the PPVs, and then proceed with the original algorithm^40^ such that steps (5) through (8) are done for every iteration (i.e. the addition of each new PPV to the model):
5. Check the direction of the effect of each PPV in the model for consistency with *a priori* assumptions (Table 3) to ensure that final models did not contradict knowledge about pollution emissions and dispersion.
6. Ensure a *p*-value of < 0.1 for each PPV.
7. Ensure that each new PPV increases the coefficient of determination (R^2^) for a LOOCV^51^.
8. Calculate a multicollinearity index called the variance inflation factor (VIF)^52^.
9. Finally, restrict the number of predictor variables in LUR model to 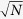, where N denotes the number of monitoring stations.
10. Check the normality of residuals using the Shapiro-Wilk test^50^.

The algorithm was programmed as a function in the R statistical package. Its details are explained in pages S7–S11, the supplemental information, and in the original paper by Amini *et al*.^40^. Models were constructed for average annual, cooler season, and warmer season concentrations of NO, NO_2_, and NOx.

To check the stability of the final LUR models, the regression coefficients for the LOOCV models were retained for all predictor variables in the final NO, NO_2_, and NOx models. The minimum, maximum, and coefficient of variation were calculated for the set of LOOCV coefficients, and models with lower variability were considered to be more stable. The spatial autocorrelations for all annual and seasonal NO, NO_2_, and NOx residuals were evaluated by calculating the global Moran’s I statistic. Values of Moran’s I range from −1.0 to 1.0, with −1.0 meaning perfect negative autocorrelation, 1.0 meaning perfect positive autocorrelation, and 0 meaning a random spatial pattern^53^.

### Regression mapping

When generating raster variables from vector data, raster cells outside of the buffer zones are returned as null (or “*NoData*” in ArcGIS). All null values for the *Traffic Surrogates, Land Use, Distance Variables, Population Density, Product Variables, and Geographic Location* variables were set to zero. The Raster Calculator in the ArcGIS Spatial Analyst Tools was used to render our final nine regression equations into maps that estimated annual and seasonal concentrations of NO, NO_2_, and NO_X_ across the study area. We established a limit of prediction for low values, defined as the minimum observed concentration divided by the square root of two. All grid cells with estimates below this limit were set to this limit. Grid cells with very high estimates were set to 120% of the maximum observed concentrations, as per Henderson *et al*.^22^ and Amini *et al*.^40^.

### Seasonality of the spatial variability

In order to evaluate the effect of season on the spatial variability in NO, NO_2_, and NOx concentrations, we assessed the correlations between annual, cooler season, and warmer estimates at 1000 locations within the study area. These were randomly selected using the Feature Class Data Management Tools in ESRI ArcMap 10.2.1 GIS (ESRI, Redlands, CA). We checked the normality of the estimate distributions with a Shapiro-Wilk test, and we calculated the Pearson or Spearman correlation depending on the results.

## Discussion

This study developed annual and seasonal LUR models for NO, NO_2_ and NOx for the Middle Eastern megacity of Tehran, Iran, using data from 23 sites in the air quality monitoring network. The models performed reasonably well for all pollutants and time periods. Because there are few comparable studies published for LMICs, the discussion will focus on the observed patterns in concentrations, and the strengths and limitations of the models.

We found that the 2010 annual NO, NO_2_, and NO_X_ concentrations were relatively high in Tehran. The mean NO concentrations (88 ppb) were more than five times higher than those reported for other large cities, such as New York (16 ppb)^54^, and the mean NO_2_ concentrations (53 ppb) were almost 2.5 times higher than the recommended WHO guideline value of 21 ppb^55^. They were also considerably higher than the 2008 concentrations reported for many comparable megacities, such as Delhi (18.8 ppb), São Paulo (24.6 ppb), Tokyo (28.7 ppb), Mexico City (29.3 ppb), Los Angeles (34.5 ppb), and Dhaka (43.3 ppb), and approaching the values in Beijing (63.8 ppb)^56^.

Overall, the concentrations of nitrogen oxides were higher in the cooler season than in the warmer season (Fig. 1). This is consistent with the findings of Matte *et al*. (2013), where NO and NO_2_ concentrations in New York were higher in winter than summer^54^, and findings of Dons *et al*. (2014) in Antwerp (Belgium)^57^. The higher concentrations during the cooler season in Tehran could be due to residential heating, which is done primarily by natural gas^36^. There are also seasonal differences in meteorological factors given the specific topographical situation of the city, including inversions and low mixing heights. This may lead to more complex spatial variability in pollutants and different exposure patterns.

When considering the R^2^, adjusted R^2^, and LOOCV R^2^ values, model performance was better in the cooler season than in the warmer season for NO_2_ and NO_X_, but the opposite was true for NO (Table 1). Regardless, several of the cooler and warmer season models shared the same predictor variables. The most predictive variables for all pollutants were surrogates of traffic impact, including distance to the traffic access control zone (DIST to TACZ) in the NO and NOx models (Table 1, and Figure S3, supplemental information). This is a high traffic zone in the middle of Tehran, with access restricted to authorized vehicles on working days. It supports the hypothesis that the major source of NO and NOx in Tehran is vehicles and traffic. The natural logarithm of distance to nearest primary school (LNDIST to PRSC) appeared in eight out of nine models. All models indicate that the primary schools tended to be located in less polluted areas (Figure S4, supplemental information).

Another important predictor was green space within buffers up to 500 meters. The negative coefficients suggest that concentrations of nitrogen oxides decreased as the green space increased, which supports the call for urban greening to improve air quality and overall health^58^. We also observed increasing nitrogen oxides with increasing elevation, but decreasing concentrations with increasing slopes. This may reflect different traffic flows through the city, where the northern and southern outskirts differ in elevation by almost 800 meters. In the cooler season, the NO concentrations were also increased in areas with higher total population density, which is consistent with the hypothesis that seasonal differences were driven by residential heating. Both the annual and warmer season mean NOx concentrations increased with higher bridge density. These are predominantly land bridges that allow one roadway to pass over another roadway, replacing roundabouts and traffic lights to control traffic flow. They are found at most intersections of major roads in Tehran, and also at many smaller intersections.

The correlations of cooler and warmer season measured concentrations across the 23 fixed sites were very high for NO and NOx, but they were reduced to 0.64 and 0.58, respectively, across the 1000 randomly-selected locations. The correlation for NO_2_ was 0.61 across the fixed sites and 0.30 across the 1000 locations. Visual inspection of the pollution maps showed some interesting seasonal differences in the spatial distributions of NO_2_. One region in the northern part of the city appeared highly polluted in the cooler season, but not in the warmer season. This region had some gaps in the monitoring data, so the validity of the model may have been compromised despite our use of Amelia (see S4 to S6, supplementary information). Overall, however, our findings suggest that epidemiologic studies based on long-term exposures should account for seasonal patterns in the spatial data.

To date, many LUR models have been developed for NO_2_ in high-income countries, mainly because NO_2_ is quite easy to measure with passive samplers^21^. However, some studies have also modeled NO and NOx^22^,^37^,^38^,^59^. In all of these studies, direct or surrogate measures of traffic have been the most predictive variables. For example, in Oslo (Norway) all oxides of nitrogen were modelled using elevation, length of large roads in a 100 m buffer, length of medium roads in a 250 m buffer, and length of small roads in a 1000 m buffer based on 80 measurement locations^37^. In Tehran, the models were mostly driven by distance to the traffic access control zone and the presence of bridges in a 400 m buffer. Su *et al*. (2009) conducted a study to estimate NO, NO_2_, and NOx using 201 locations in Los Angeles (California) for two seasons. They found that traffic volume, truck routes, road networks, land use, greenness, and slope gradients were the most predictive variables^38^. We found similar explanatory variables in Tehran using data from 23 regulatory monitoring locations. However, the magnitude and ranking of the R^2^ values in Los Angeles were more similar to those in Oslo, with 81% for NO, 85% for NOx, and 86% for NO_2_^38^. Results from Vancouver (Canada) are also consistent with our findings in Tehran, with traffic variables, elevation, geographic coordinates, and total population within 2500 m buffer radius driving the models^22^. In Montreal (Canada) Gilbert *et al*. (2005) found that distance to highways, lengths of roads within buffers of 100–500 m, open space, and population density within a radius of 2000 m were the most predictive variables for NO_2_, and the best-fitting model had an R^2^ of 0.54^60^.

The use of fixed site monitor locations to develop the LUR models can be both a strength and a limitation. Readily-available data from validated instruments allows academics and government agencies to regularly model the spatial variability in air pollutants with minimal additional costs. However, the locations for fixed monitoring networks are generally chosen by criteria that may not optimize their ability to capture the variability necessary for spatial modelling^28^. Although we did not evaluate whether 23 measurement sites are sufficient to reliably model spatial variability in a megacity such as Tehran^27^, our future work will examine this question in more detail.

Another limitation is that some predictor variables could not be assigned a direction of effect *a priori* due to lack of previous knowledge or other studies. This, in turn, might have caused inconsistent effects of variables in the regression models. These variables include urban facilities, sensitive areas, such as military and protected government areas, other land use variables, distance to hazardous facilities areas, distance to food shops, distance to airports, distance to health and ambulance services, elevation, and slope gradients. Therefore, we suggest conducting further studies in Tehran to better specify the impact of these areas on air pollution concentrations.

## Conclusions

We found significant seasonal differences in the spatial variation of nitrogen oxides in Tehran, especially NO_2_. However, the small number of measurement sites in our study might affect these findings. Examples of LUR models are rare in LMICs, and these results are relevant for the next generation of exposure assessment, population-based health research, and policy-making in such contexts. In addition, this work establishes a benchmark for future air pollution modeling in Tehran. Overall, our models performed relatively well. Our next step is to evaluate whether a larger number of monitoring sites selected with a strict algorithm produces different results and/or different conclusions about the spatial patterns reported here.

## Additional Information

**How to cite this article**: Amini, H. *et al*. Annual and seasonal spatial models for nitrogen oxides in Tehran, Iran. *Sci. Rep.* **6**, 32970; doi: 10.1038/srep32970 (2016).

## Supplementary Material

Supplementary Information

## Figures and Tables

**Table 1:** Final land use regression models for annual and seasonal concentrations of NO, NO_2_ and NO_X_ in Tehran, Iran.

Response	Equation (variables are ordered by partial R^2^)	R^2^	Adjusted R^2^	LOOCV^a^ R^2^	Highest Variance Inflation Factor (variable)	*p*-value	RMSE^b^	Measured Response^c^
Log Annual NO	1.53 − 1.4e-04 × **DIST to TACZ** + 6.9e-01 × **LNDIST to PRSC** − 3.1e-06 × **GRS.500** − 4.4e-02 × **SLP** − 3.0e-05 × URF.100	0.78	0.71	0.66	1.8 (LNDIST to PRSC)	<0.001	32.1	88 (23–312)
Log Cooler Season NO	1.92 + 5.1e-01 × **LNDIST to PRSC** + 4.7e-05 × TPDC.2500 − 1.1e-04 × DIST to BST − 3.2e-04 × DIST to PST − 2.1e-06 × **GRS.400**	0.69	0.60	0.53	1.5 (TPDC.2500)	<0.001	38.6	117 (30–358)
Log Warmer Season NO	0.68 − 1.5e-04 × **DIST to TACZ** + 7.4e-01 × **LNDIST to PRSC** − 6.3e-02 × **SLP** − 2.0e-06 × **GRS.500** + 9.4e-04 × DIST to GRS	0.72	0.64	0.59	2.2 (SLP)	<0.001	36.9	60 (17–268)
Log Annual NO_2_	2.9 + 1.1e-05 × **OFIC.300** − 1.5e-04 × **DIST to SNS** + 1.7e-01 × **LNDIST to PRSC** + 2.2e-05 × **OTHR.300**	0.69	0.62	0.57	1.3 (LNDIST to PRSC)	<0.001	9.9	53 (22–96)
(Log Cooler Season NO_2_)^3^	−5.7e + 01 + 5.9e-04 × **OFIC.300** + 1.1e-01 × × ELEV − 4.1e-03 × DIST to AIR + 8.3e-04 × **OTHR.400** − 1.5e-03 × ARD.100	0.75	0.68	0.58	3.8 (DIST to AIR)	<0.001	9.2	62 (21–103)
(Log Warmer Season NO_2_)^−1^	3.3e-01 − 6.8e-07 × **OFIC.300** + 1.2e-05 × **DIST to SNS** − 1.0e-02 × **LNDIST to PRSC**	0.64	0.58	0.51	1.2 (LNDIST to PRSC)	<0.001	10.2	45 (23–89)
(Log Annual NO_X_)^−2^	9.2e-02 + 2.0e-06 × **DIST to TACZ** − 6.5e-03 × **LNDIST to PRSC** + 2.6e-05 × DIST to OFIC − 3.2e-03 × **LNDIST to HZRFAC** − 1.2e-03 × **BGD.400**	0.71	0.62	0.58	1.7 (DIST to OFIC)	<0.001	52.7	142 (66–385)
(Log Cooler Season NO_X_)^−1^	2.9e-01 + 5.8e-06 × × **DIST to TACZ** − 1.1e-02 × **LNDIST to HZRFAC** + 1.6e-06 × URF.100 + 8.8e-08 × GRS.400 − 9.2e-03 × **LNDIST to PRSC**	0.79	0.73	0.63	1.9 (DIST to TACZ)	<0.001	37.1	180 (76–435)
(Log Warmer Season NO_X_)^−4^	7.1e-03 − 8.1e-04 × **LNDIST to PRSC** + 1.3e-07 × **DIST to TACZ** − 4.0e-04 × (OFIC.100)^0.1^ − 1.3e-04 × × **BGD.400** + 3.4e-05 × SLP	0.61	0.50	0.42	1.9 (LNDIST to PRSC)	0.004	44.8	105 (49–336)
Radius variable types included in the models were:			The log-linear distance variables included in the models were:					
**GRS** = green space area			** LNDIST to HZRFAC** = log distance to hazardous facilities					
**OFIC** = official/commercial land use area			** LNDIST to PRSC** = log distance to the nearest primary school					
**OTHR** = other land use area								
**URF** = urban facilities area			Other variable included in the models were:					
**ARD** = arid/undeveloped area			** BGD** = bridge length in a buffer radii divided by distance to the bridges					
The linear distance variables included in the models were:			** ELEV** = elevation					
**DIST to AIR** = distance to airport or air cargo facilities			** SLP** = slope					
**DIST to BST** = distance to bus terminal			** TPDC** = population density excluding unemployed and children <5 years					
**DIST to GRS** = distance to green space area								
**DIST to SNS** = distance to sensitive area			For variables of the form **XXX.YYY** the **XXX** indicates the variable type, and the **YYY** indicates the buffer size, in meters.					
**DIST to OFIC** = distance to official/commercial area								
**DIST to PST** = distance to petrol stations								
**DIST to TACZ** = distance to the traffic access control zone								

Variables in bold highlight consistencies between models for the same pollutant—see SI, Tables S2–S7 for full description of each model.

^a^Leave one out cross validation;

^b^Root mean square error = 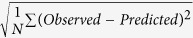;

^c^Mean (min–max); note that the units are ppb. The *p*-values of underlined variables are ≤0.001; The *p*-values of dotted-underlined variables are ≤0.01; The *p*-values of wave-underlined variables are ≤0.05.

**Table 2:** The percentage of predicted grid cells out of >24 million cells in the study area that either enlarged to the quantification limit or truncated to 120% of the maximum observed concentrations by 2010 LUR models in Tehran, Iran.

Action	Pollutant	Annual	Cooler season	Warmer season
Enlarged	NO	8.9%	11.3%	11.4%
	NO_2_	0.4%	2.1%	0.2%
	NOx	16.0%	12.1%	0.5%
Truncated	NO	0.1%	0.0%	1.5%
	NO_2_	4.6%	5.3%	2.0%
	NOx	1.0%	1.3%	0.9%

**Table 3:** The spatial predictor variables, assumed directions of their effects on pollutant concentrations, raw inputs, and the procedures for generating them.

Variable class (N variables)	Description	Variable sub-class (N variables)	Buffer radii (m)	Assumed effect	Input file type & source, and procedure
Traffic Surrogates (26)	Total length of road types and bridges (m)	ST = streets (5) HW = highways (5) RDa = major roads (7) RDb = all roads (7) BG = bridges (2)	100–500 100–1000 400–500	+++++	Polyline format, JICA and CEST^a^ (a) Convert polyline files into raster files with 5 m pixel size (b) Use *Neighborhood, Focal Statistics*^b^ to sum the number of road pixels within the search radii (c) Multiply the result by 5
Land Use (50)	Total area of 10 LU types (m^2^)	RES = residential (5) GRS = green space (5) URF = urban facilities (5) IND = industrial/workshop (5) OFIC = official/commercial (5) TRS = transportation (5) SNS = sensitive areas (5) AGR = agriculture (5) ARD = arid/undeveloped (5) OTHR = other (5)	100–500	− − ?^c^ + + + ?^c^ − − ?^c^	Polygon format, JICA and CEST^a^ (a) Convert land use polygons into 10 raster files for RES, GRS, URF, IND, OFIC, TRS, SNS, AGR, ARD, and OTHR with 5 m pixel size (b) Use *Neighborhood, Focal Statistics*^b^ to sum the number of land use type pixels within the search radii (c) Multiply the result by 25
Distance Variables (60)	Distance (DIST) and log distance (LNDIST) to various features (m)	DIST and LNDIST to: All Traffic Surrogate and Land Use variables (30) FWY = freeways (2) TACZ = traffic access control zone (2) TACAP = TACZ in critical air pollution conditions (2) SPLND = sport land (2) PRSC = primary school (2) SCSC = high school (2) PST = petrol stations (2) PRK = park (2) MSQ = mosque (2) HZRFAC = hazardous facility (2) FV = various food shops (2) BST = bust terminal (2) AIR = airport or air cargo facilities (2) AMB = ambulance service (2)	N/A	Opposite of above − − − + + + − + + ?^c^ ?^c^ − ?^c^ ?^c^	Raster format, calculated from raw files of JICA and CEST^a^ Use *Spatial Analyst, Distance, Straight Line* to produce DIST variables; use *Spatial Analyst, Raster Calculator* to produce LNDIST variables
Population Density (22)	Density of population (persons per km^2^)	PD = total (11) TPDC = PD excluding unemployed and children <5 years (11)	500–3000	+ +	Polygon format, JICA and CEST^a^ a) Convert census polygons to centroids; assign each centroid the population count of the polygon from which it was derived (b) Use *Kernel Density* to estimate the values within each radius.
Product or Ratio Variables (52)	Integrated products of the traffic surrogates and distance variable classes	STD = ST/DISTST (5) HWD = HW/DISTHW (5) STSQD = ST/sq(DISTST) (5) HWSQD = HW/sq(DISTHW) (5) RDaD = RDa/DISTRDa (7) RDbD = RDb/DISTRDb (7) RDaSQD = RDa/sq(DISTRDa) (7) RDbSQD = RDb/sq(DISTRDb) (7) BGD = BG/DISTBG (2) BGSQD = BG/sq(DISTBG) (2)	100–500 100–1000 400–500	++++++++++	Raster format, calculated from raw files of JICA and CEST^a^ Use *Spatial Analyst, Raster Calculator* to create Product Variables
Geographic Location (2)	Physical location	ELEV = elevation (m) SLP = slope (degree)	N/A	?^c^ ?^c^	Digital Elevation Model (DEM), NCCI^d^ Use *Spatial Analyst, Surface Analysis* to create slope from DEM

Modified from ref. 40 with permission from Elsevier.

^a^Japan International Cooperation Agency and Center for Earthquake and Environmental Studies of Tehran.

^b^Features of the Spatial Analyst Tools to ESRI’s ArcMap 10.2.1 GIS (ESRI, Redlands, CA).

^c^No *a priori* assigned because no effect could be assumed.

^d^National Cartographic Center of Iran.

## References

[b1] Künzli N. . Public-health impact of outdoor and traffic-related air pollution: a European assessment. Lancet 356, 795–801 (2000). 11022926 10.1016/S0140-6736(00)02653-2

[b2] Kaufman J. D. . Association between air pollution and coronary artery calcification within six metropolitan areas in the USA (the Multi-Ethnic Study of Atherosclerosis and Air Pollution): a longitudinal cohort study. Lancet 388, 696–704 (2016). 27233746 10.1016/S0140-6736(16)00378-0 PMC5019949

[b3] Brauer M. . Ambient air pollution exposure estimation for the Global Burden of Disease 2013. Environ. Sci. Technol. 50, 79–88 (2016). 26595236 10.1021/acs.est.5b03709

[b4] Künzli N., Joss M. K. & Gintowt E. Global standards for global health in a globalized economy! Int. J. Public Health 60, 757–759 (2015). 26292652 10.1007/s00038-015-0729-0

[b5] Forouzanfar M. H. . Global, regional, and national comparative risk assessment of 79 behavioural, environmental and occupational, and metabolic risks or clusters of risks in 188 countries, 1990–2013: a systematic analysis for the Global Burden of Disease Study 2013. Lancet 386, 2287–2323 (2015). 26364544 10.1016/S0140-6736(15)00128-2 PMC4685753

[b6] Poursafa P. . Trends in health burden of ambient particulate matter pollution in Iran, 1990–2010: findings from the global burden of disease study 2010. Environ. Sci. Pollut. Res. 22, 18645–18653 (2015). 10.1007/s11356-015-5545-9 26490896

[b7] Gharehchahi E. . Health impact assessment of air pollution in Shiraz, Iran: a two-part study. J. Environ. Health Sci. Eng 11, 8, doi: 10.1186/2052-336x-11-11 (2013). 24499576 PMC3776287

[b8] Institute for Health Metrics and Evaluation (IHME). GBD Compare. Seattle, WA: IHME, University of Washington, 2015. Available from http://vizhub.healthdata.org/gbd-compare. (Accessed [July 09, 2016]).

[b9] Giorgini P. . Air pollution exposure and blood pressure: an updated review of the literature. Curr. Pharm. Design 22, 28–51 (2016). 10.2174/1381612822666151109111712 26548310

[b10] Fuks K. B. . Arterial blood pressure and long-term exposure to traffic-related air pollution: an analysis in the European Study of Cohorts for Air Pollution Effects (ESCAPE). Environ. Health Perspect. 122, 896–905 (2014). 24835507 10.1289/ehp.1307725 PMC4154218

[b11] Eze I. C. . Association between ambient air pollution and diabetes mellitus in Europe and North America: systematic review and meta-analysis. Environ. Health Perspect. 123, 381–389 (2015). 25625876 10.1289/ehp.1307823 PMC4421762

[b12] Hansen A. B. . Long-term exposure to fine particulate matter and incidence of diabetes in the Danish Nurse Cohort. Environ. Int. 91, 243–250 (2016). 26989812 10.1016/j.envint.2016.02.036

[b13] Eze I. C. . Long-Term Exposure to Ambient Air Pollution and Metabolic Syndrome in Adults. PloS One 10, e0130337, doi: 10.1371/journal.pone.0130337 (2015). 26103580 PMC4478007

[b14] Heydarpour P. . Potential impact of air pollution on multiple sclerosis in Tehran, Iran. Neuroepidemiology 43, 233–238 (2014). 25501708 10.1159/000368553

[b15] Oudin A. . Traffic-Related Air Pollution and Dementia Incidence in Northern Sweden: A Longitudinal Study. Environ. Health Perspect. 124, 306–312 (2015). 26305859 10.1289/ehp.1408322 PMC4786976

[b16] Yan W., Yun Y., Ku T., Li G. & Sang N. NO2 inhalation promotes Alzheimer’s disease-like progression: cyclooxygenase-2-derived prostaglandin E2 modulation and monoacylglycerol lipase inhibition-targeted medication. Sci Rep 6, doi: 10.1038/srep22429 (2016). PMC4772479 26928013

[b17] LaKind J. S. . A proposal for assessing study quality: Biomonitoring, Environmental Epidemiology, and Short-lived Chemicals (BEES-C) instrument. Environ. Int. 73, 195–207 (2014). 25137624 10.1016/j.envint.2014.07.011 PMC4310547

[b18] Jerrett M. . A review and evaluation of intraurban air pollution exposure models. J. Expo. Sci. Environ. Epidemiol. 15, 185–204 (2005). 10.1038/sj.jea.7500388 15292906

[b19] Zou B., Wilson J. G., Zhan F. B. & Zeng Y. Air pollution exposure assessment methods utilized in epidemiological studies. J. Environ. Monit. 11, 475–490 (2009). 19280026 10.1039/b813889c

[b20] Tashayo B. & Alimohammadi A. Modeling urban air pollution with optimized hierarchical fuzzy inference system. Environ. Sci. Pollut. Res., doi: 10.1007/s11356-016-7059-5 (2016). 27378222

[b21] Hoek G. . A review of land-use regression models to assess spatial variation of outdoor air pollution. Atmos. Environ. 42, 7561–7578 (2008).

[b22] Henderson S. B., Beckerman B., Jerrett M. & Brauer M. Application of land use regression to estimate long-term concentrations of traffic-related nitrogen oxides and fine particulate matter. Environ. Sci. Technol. 41, 2422–2428 (2007). 17438795 10.1021/es0606780

[b23] Basagaña X. . Measurement error in epidemiologic studies of air pollution based on land-use regression models. Am. J. Epidemiol. 178, 1342–1346 (2013). 24105967 10.1093/aje/kwt127

[b24] Zou B. . Performance comparison of LUR and OK in PM2. 5 concentration mapping: a multidimensional perspective. Sci Rep 5, doi: 10.1038/srep08698 (2015). PMC4346829 25731103

[b25] Rivera M. . Association between long-term exposure to traffic-related air pollution and subclinical atherosclerosis: the REGICOR study. Environ. Health Perspect. 121, 223–230 (2013). 23384708 10.1289/ehp.1205146 PMC3569680

[b26] de Hoogh K. . Development of West-European PM 2.5 and NO2 land use regression models incorporating satellite-derived and chemical transport modelling data. Environ. Res. 151, 1–10, doi: 10.1016/j.envres.2016.07.005 (2016). 27447442

[b27] Basagaña X. . Effect of the number of measurement sites on land use regression models in estimating local air pollution. Atmos. Environ. 54, 634–642 (2012).

[b28] Kanaroglou P. S. . Establishing an air pollution monitoring network for intra-urban population exposure assessment: A location-allocation approach. Atmos. Environ. 39, 2399–2409 (2005).

[b29] Ross Z., Jerrett M., Ito K., Tempalski B. & Thurston G. D. A land use regression for predicting fine particulate matter concentrations in the New York City region. Atmos. Environ. 41, 2255–2269 (2007).

[b30] Moore D., Jerrett M., Mack W. & Künzli N. A land use regression model for predicting ambient fine particulate matter across Los Angeles, CA. J. Environ. Monit. 9, 246–252 (2007). 17344950 10.1039/b615795e

[b31] Gulliver J., de Hoogh K., Fecht D., Vienneau D. & Briggs D. Comparative assessment of GIS-based methods and metrics for estimating long-term exposures to air pollution. Atmos. Environ. 45, 7072–7080 (2011).

[b32] Meng X. . A land use regression model for estimating the NO 2 concentration in shanghai, China. Environ. Res. 137, 308–315 (2015). 25601733 10.1016/j.envres.2015.01.003

[b33] Wark K. & Warner C. F. Air pollution: its origin and control. (Harper & Row, 1981).

[b34] Seinfeld J. H. & Pandis S. N. Atmospheric chemistry and physics: from air pollution to climate change. (John Wiley & Sons, 2012).

[b35] Kerckhoffs J. . A national fine spatial scale land-use regression model for ozone. Environ. Res. 140, 440–448 (2015). 25978345 10.1016/j.envres.2015.04.014

[b36] Amini H. . National and sub-national Environmental Burden of Disease in Iran from 1990 to 2013-study profile. Arch. Iran. Med. 17, 62 (2014). 24444065

[b37] Madsen C. . Modeling the intra-urban variability of outdoor traffic pollution in Oslo, Norway—A GA 2 LEN project. Atmos. Environ. 41, 7500–7511 (2007).

[b38] Su J. G. . Predicting traffic-related air pollution in Los Angeles using a distance decay regression selection strategy. Environ. Res. 109, 657–670 (2009). 19540476 10.1016/j.envres.2009.06.001 PMC3656661

[b39] Eeftens M. . Development of land use regression models for nitrogen dioxide, ultrafine particles, lung deposited surface area, and four other markers of particulate matter pollution in the Swiss SAPALDIA regions. Environ. Health 15, doi: 10.1186/s12940-016-0137-9 (2016). PMC4835865 27089921

[b40] Amini H. . Land use regression models to estimate the annual and seasonal spatial variability of sulfur dioxide and particulate matter in Tehran, Iran. Sci. Total Environ. 488, 343–353 (2014). 24836390 10.1016/j.scitotenv.2014.04.106

[b41] Chen L. . A land use regression for predicting NO2 and PM10 concentrations in different seasons in Tianjin region, China. J. Environ. Sci. 22, 1364–1373 (2010). 10.1016/s1001-0742(09)60263-1 21174967

[b42] Ryan P. H. & LeMasters G. K. A review of land-use regression models for characterizing intraurban air pollution exposure. Inhal. Toxicol. 19, 127–133 (2007). 17886060 10.1080/08958370701495998 PMC2233947

[b43] Amini H., Taghavi-Shahri S.-M., Naddafi K., Nabizadeh R. & Yunesian M. Correlation of air pollutants with land use and traffic measures in Tehran, Iran: A preliminary statistical analysis for land use regression modeling. J. Adv. Environ. Health Res. 1, 1–8 (2013).

[b44] Honaker J., King G. & Blackwell M. Amelia II: A program for missing data. J. Stat. Softw. 45, 1–47 (2011).

[b45] Roorda-Knape M. C. . Air pollution from traffic in city districts near major motorways. Atmos. Environ. 32, 1921–1930 (1998).

[b46] Janssen N. A., van Vliet P. H., Aarts F., Harssema H. & Brunekreef B. Assessment of exposure to traffic related air pollution of children attending schools near motorways. Atmos. Environ. 35, 3875–3884 (2001).

[b47] Zhu Y., Hinds W. C., Kim S., Shen S. & Sioutas C. Study of ultrafine particles near a major highway with heavy-duty diesel traffic. Atmos. Environ. 36, 4323–4335 (2002).

[b48] Forsberg B. . Comparative health impact assessment of local and regional particulate air pollutants in Scandinavia. Ambio 34, 11–19 (2005). 15789513

[b49] JICA and CEST. The study on seismic micro-zoning of the greater Tehran area in the Islamic Republic of Iran. A report from Japan International Cooperation Agency (JICA) and Center for Earthquake and Environmental Studies of Tehran (CEST), Tehran Municipality, Tehran, Iran. Available via: http://www.vojoudi.com/earthquake/jica/. Accessed April 17, 2016 [In Persian]. (2000).

[b50] Clayton D., Hills M. & Pickles A. Statistical models in epidemiology. Vol. 161 (IEA, 1993).

[b51] Efron B. & Gong G. A leisurely look at the bootstrap, the jackknife, and cross-validation. Am. Stat. 37, 36–48 (1983).

[b52] O’brien R. M. A caution regarding rules of thumb for variance inflation factors. Qual. Quant. 41, 673–690 (2007).

[b53] Moran P. A. Notes on continuous stochastic phenomena. Biometrika 37, 17–23 (1950). 15420245

[b54] Matte T. D. . Monitoring intraurban spatial patterns of multiple combustion air pollutants in New York City: Design and implementation. J. Expo. Sci. Environ. Epidemiol. 23, 223–231 (2013). 23321861 10.1038/jes.2012.126

[b55] WHO. WHO Air quality guidelines for particulate matter, ozone, nitrogen dioxide and sulfur dioxide. Geneva (2005). 34662007

[b56] Gurjar B., Butler T., Lawrence M. & Lelieveld J. Evaluation of emissions and air quality in megacities. Atmos. Environ. 42, 1593–1606 (2008).

[b57] Dons E. . Land use regression models as a tool for short, medium and long term exposure to traffic related air pollution. Sci. Total Environ. 476, 378–386 (2014). 24486493 10.1016/j.scitotenv.2014.01.025

[b58] Bowler D. E., Buyung-Ali L., Knight T. M. & Pullin A. S. Urban greening to cool towns and cities: A systematic review of the empirical evidence. Landsc. Urban Plan. 97, 147–155 (2010).

[b59] Beelen R., Hoek G., Fischer P., van den Brandt P. A. & Brunekreef B. Estimated long-term outdoor air pollution concentrations in a cohort study. Atmos. Environ. 41, 1343–1358 (2007).

[b60] Gilbert N. L., Goldberg M. S., Beckerman B., Brook J. R. & Jerrett M. Assessing spatial variability of ambient nitrogen dioxide in Montreal, Canada, with a land-use regression model. J. Air Waste Manage. Assoc. 55, 1059–1063 (2005). 10.1080/10473289.2005.10464708 16187576

