# Supplementary material for: Annual and seasonal spatial models for nitrogen oxides in Tehran, Iran
Source: Sci Rep. 2016 Sep 13;6:32970. doi: 10.1038/srep32970 (PMC5020732; doi:10.1038/srep32970)
Supplement: Supplementary Information [file srep32970-s1.pdf]

Supplemental Information

Annual and seasonal spatial models for nitrogen oxides in Tehran, Iran

Heresh Amini[1,2], Seyed Mahmood Taghavi-Shahri[3,4], Sarah B. Henderson[5,6], Vahid Hosseini[7], Hossein Hassankhany[8], Maryam Naderi[8], Solmaz Ahadi[8], Christian Schindler[1,2], Nino Künzli[1,2], Masud Yunesian[9,*]

[1]Department of Epidemiology and Public Health, Swiss Tropical and Public Health Institute, Basel, Switzerland

[2]University of Basel, Basel, Switzerland

[3]Research Center for Environmental Pollutants, Qom University of Medical Sciences, Qom, Iran

[4]Department of Epidemiology and Biostatistics, School of Public Health, Isfahan University of Medical Sciences, Isfahan, Iran

[5]Environmental Health Services, British Columbia Centre for Disease Control, Vancouver, Canada

[6]School of Population and Public Health, University of British Columbia, Vancouver, Canada

[7]Mechanical Engineering Department, Sharif University of Technology, Tehran, Iran

[8]Tehran Air Quality Control Co., Tehran Municipality, Tehran, Iran

[9]Center for Air Pollution Research (CAPR), Institute for Environmental Research (IER), Tehran University of Medical Sciences, Tehran, Iran

***Correspondence:**
Prof. Masud Yunesian, MD, PhD
Center for Air Pollution Research (CAPR)
Institute for Environmental Research (IER)
Tehran University of Medical Sciences, Tehran, Iran
PO Box: 14155-6446
Tel: +98 21 8898 7379
Fax: +98 21 8163 3623
E-mail address: yunesian@tums.ac.ir

The Supplemental Information Contains:

25 Pages

4 Figures

11 Tables

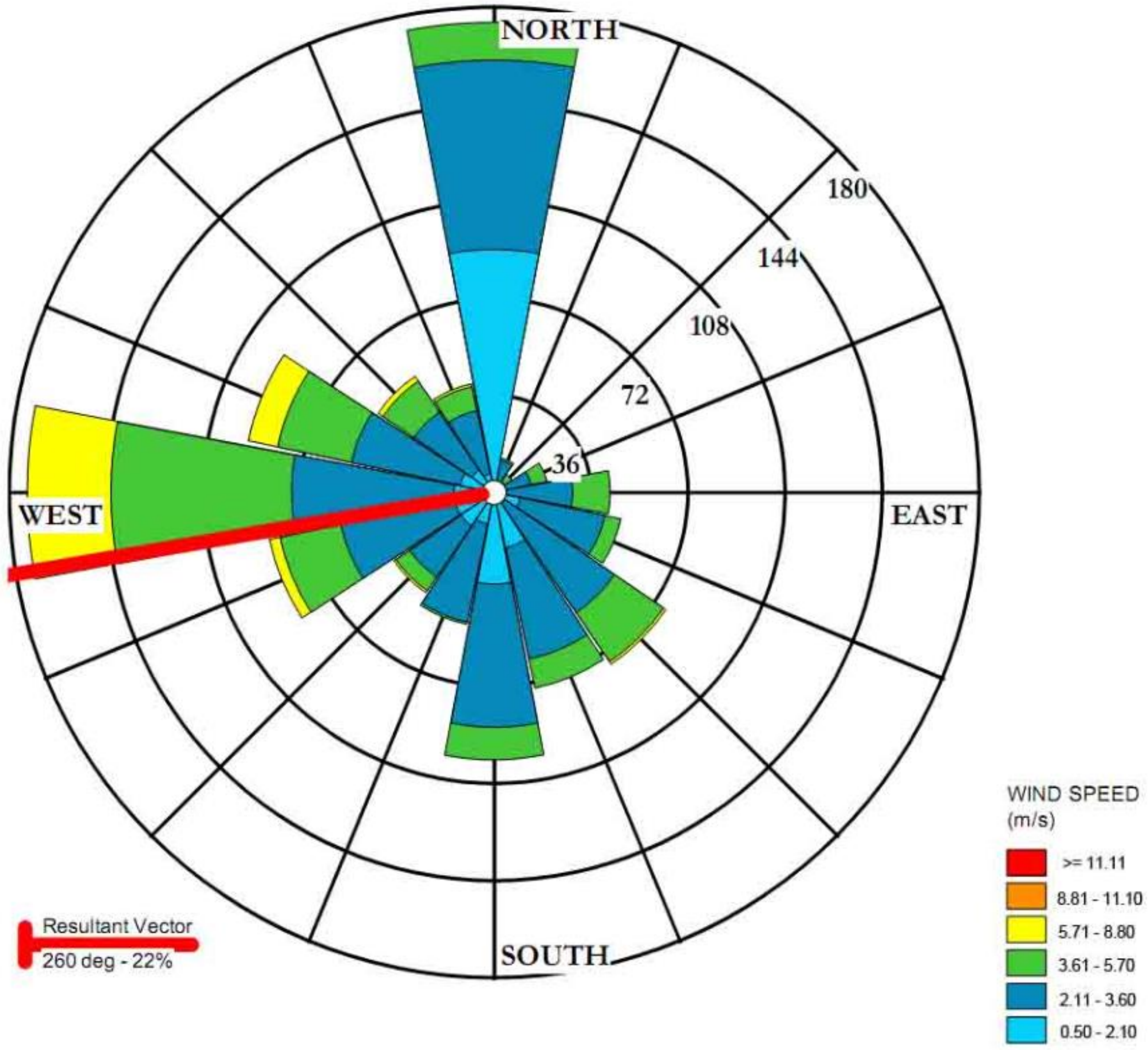

Figure S1. Wind-rose diagram for the Mehrabad International Airport meteorology station, indicating the wind is predominantly from the west and north.

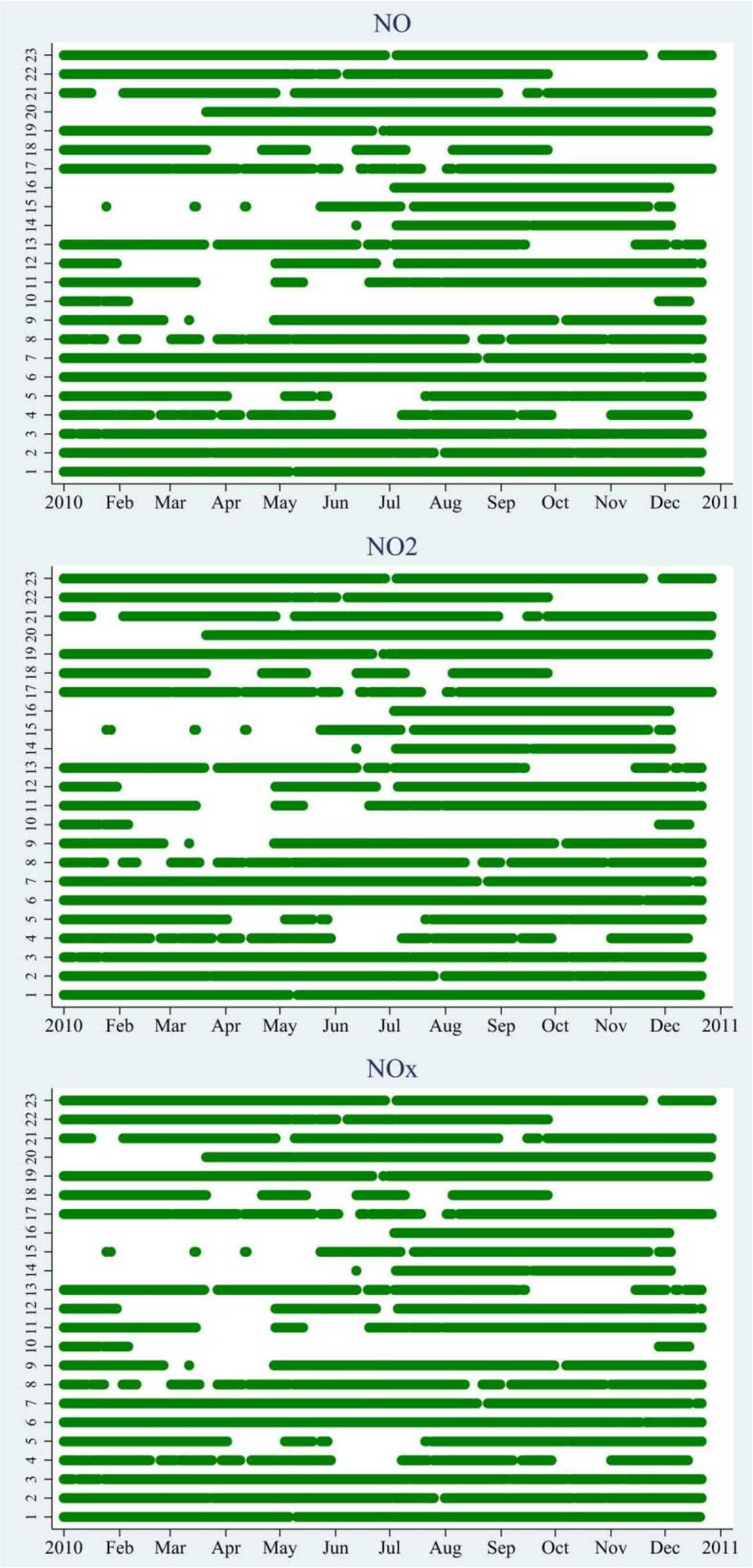

Figure S2. Details of the data available for each air quality monitoring station. The x-axis shows time in months and y-axis shows available data for each of stations out of 23. The green color for each station shows that the data were available and the white areas are missing data.

Imputation of missing data

The Amelia program was used for imputation of the missing data (Figure S2 shows missing NO, NO_2 and NO_X measurements) [1]. The program uses a new expectation-maximization algorithm with bootstrapping to impute missing values and return a complete dataset. We provided the program with all available hourly concentrations from the different stations, along with the month, day, and hours of measurement. In order to evaluate the precision of the missing data estimates we ran the Amelia program 10 times for each pollutant, and calculated the resulting 10 annual and seasonal averages for each monitoring station. Next, we calculated the coefficient of variation (CV) between the 10 annual and seasonal means. If the CV was small (less than about 5%), the estimates were considered acceptable. If not, the station was removed from further analyses because of the low precision of the annual and/or seasonal estimates.

The maximum CVs for the 23 stations used to model NO ranged from 3.9% for the annual to 4.5% for the cooler season. The maximum CVs for the 23 stations used to model NO_2 ranged from 3.8% for the warmer season to 5.5% for the cooler season. For 23 NO_X stations, the maximum CVs were 2.9% for the warmer season to 3.2% for the cooler season. The CV averages for NO stations ranged from 0.8% for the annual to 1.1% for the warmer season. The CV averages for NO_2 stations ranged from 0.7% for the warmer season to 1.0% for the cooler season. The CV averages for NO_X stations ranged from 0.7% for the annual to 0.8% for the cooler and warmer seasons (Table S1).

Table S1. The coefficient of variation (CV) for the mean annual, cooler season, and warmer season in 10 times missing data imputation for checking reliability of the estimates.

	CVs for NO (%)			CVs for NO_2 (%)			CVs for NO_X (%)		
Station	Annual	Cooler Season	Warmer Season	Annual	Cooler Season	Warmer Season	Annual	Cooler Season	Warmer Season
1	0.2	0.2	0.4	0.1	0.1	0.1	0.1	0.1	0.2
2	0.3	0.3	0.3	0.1	0.1	0.1	0.1	0.2	0.2
3	0.3	0.4	0.3	0.1	0.2	0.1	0.2	0.3	0.2
4	0.8	1.2	2.0	0.5	1.0	0.3	0.6	0.7	1.1
5	0.4	0.3	1.6	0.2	0.1	0.4	0.4	0.2	1.1
6	0.1	0.2	0.2	0.1	0.2	0.1	0.1	0.1	0.1
7	0.3	0.3	1.0	0.2	0.2	0.3	0.2	0.2	0.2
8	0.4	0.6	0.5	0.3	0.4	0.2	0.4	0.6	0.3
9	0.2	0.3	0.6	0.1	0.2	0.1	0.2	0.2	0.4
10	1.2	1.0	1.8	1.6	1.1	2.3	1.8	1.3	2.7
11	0.4	0.4	1.3	0.1	0.1	0.2	0.2	0.2	0.6
12	0.6	0.6	0.9	0.3	0.5	0.3	0.7	0.8	0.8
13	0.5	0.5	1.1	0.2	0.3	0.2	0.4	0.5	0.5
14	3.9	4.5	4.2	4.8	5.5	3.8	2.7	3.1	2.9
15	2.0	2.4	1.3	1.9	2.2	1.5	1.1	1.5	0.5
16	3.6	4.0	3.3	2.9	2.9	2.9	3.0	3.2	2.9
17	0.4	0.1	1.5	0.4	0.1	1.2	0.4	0.1	1.4
18	1.0	1.4	0.8	1.4	2.1	1.0	1.0	1.2	0.8
19	0.1	0.1	0.2	0.0	0.1	0.1	0.1	0.1	0.2
20	0.5	1.0	0.1	1.3	1.9	0.2	0.7	1.2	0.1
21	0.2	0.2	0.5	0.2	0.2	0.4	0.3	0.2	0.5
22	0.7	1.0	0.3	1.4	2.2	0.4	0.8	1.3	0.4
23	0.2	0.3	0.3	0.2	0.3	0.1	0.1	0.2	0.2

Model development and diagnostics

We developed a systematic algorithm that considered 10 key pieces of information:

(1) Take the log transformation of the response variable
(2) Check for normality using the Shapiro-Wilk test [2]
(3) Apply a power transformation if not normally distributed
(4) Linearize the relationships between the transformed variables and the PPVs using log and power transformations on the PPVs, and then proceed with the original algorithm [3] such that steps (5) through (8) are done for every iteration (i.e. the addition of each new PPV to the model):
(5) Check the direction of the effect of each PPV in the model for consistency with *a priori* assumptions (Table 1) to ensure that final models did not contradict knowledge about pollution emissions and dispersion
(6) Ensure a p-value of < 0.1 for each PPV
(7) Ensure that each new PPV increases the coefficient of determination (R^2) for a leave-one-out cross-validation (LOOCV)[4]
(8) Calculate a multicollinearity index called the variance inflation factor (VIF)[5]
(9) Finally, restrict the number of predictor variables in LUR model to $\sqrt{N}$, where N denotes the number of monitoring stations
(10) Check the normality of residuals using the Shapiro-Wilk test.

The rationale for step 1 to 3

We decided to first log-transform the response variable data in step 1 with the benefit that the predictions get non-negative values—though it could also somewhat resolve the challenge of having a response variable that is not normally distributed (step 2). Finally, we tried to use power transformations to have a normally distributed response variable (step 3). Though in general it is required that the residuals or errors should be normally distributed in a linear regression analysis

(see step 10), once the response variable data are not normally distributed, most likely the residuals might also not have a normal distribution [6]. We checked the normality assumption by Shapiro-Wilk test [2].

The rationale for step 4

In order to linearize relationship of each explanatory variable with transformed response variable of stage 3, we tried various powers for each predictor variable to find ones that produce most correlation between this power transformation of predictor and the results of stage 3. This step caused inclusion of a variable (OFIC.100 = official/commercial land use areas in buffer 100 m) with the power of 0.1 in the final model of warmer season NOx.

The rationale for step 5

There are two approaches to LUR model building. One approach favors the most predictive model and the other approach favors the most easily interpreted model. The two are not, necessarily, mutually exclusive. In the first approach, the most predictive variables are kept regardless of their sign. We did not feel that this was the right approach for Tehran, because we wanted to be able to readily explain the modeling results to the epidemiologists who will be using them in future. Thus, we used the second approach where variables are only retained in the model if their coefficients are consistent with *a priori* assumptions about the direction of the effect (e.g. decreasing pollutant concentrations with increasing distance from traffic sources). Several other studies have followed this approach, including Henderson et al. (2007) [7], Hoek et

al. (2010) [8], Eeftens et al. (2012) [9], Gonzales et al. (2012) [10], Beelen et al. (2013) [11], de Hoogh et al. (2013) [12], Abernethy et al. (2013) [13], and Gulliver et al. (2013) [14]. We made this choice based on our situation in Tehran, to ensure that any associated epidemiologic results to be easily interpreted by policy makers.

The rationale for step 6

Some LUR studies have observed that insignificant predictors (*p*-values > 0.1) can increase the total R^2 of the regression equation [9,10,15,16]. Thus, to prevent the inclusion of such variables in our models, we set another criterion in the sixth piece of the algorithm to include only those variables with significant *p*-values ($p < 0.1$). The $p < 0.1$ was selected because it is widely applied in the LUR community [7,9].

The rationale for step 7

A recent analysis from Girona (Spain) demonstrated that LUR models developed from fewer sites had higher model R^2 values, lower LOOCV R^2 values, and different predictive variables than models developed from more sites [17]. To account for this, we designed a model-building algorithm that selected variables based on the improvements to the LOOCV R^2 value instead of the model R^2 or adjusted R^2. Model R^2 is a measure of internal validity of the model while LOOCV R^2 is a measure of external validity, thus, a more appropriate measure for model selection [18]. We believe this method, especially for study areas with small number of sites, leads to the generation of models with high R^2 and LOOCV R^2 values, as well as generation of temporal models with an internally consistent set of potentially predictive variables.

The rationale for step 8

The VIF is the reciprocal of *Tolerance,* and both are multicollinearity indices. The VIF is calculated as the following equation:

$$VIF = \frac{1}{1 - R_i^2}$$

Where $1 - R_i^2$ is the tolerance and R_i^2 denotes the proportion of variance in the *i*th predictor, which is correlated with the other predictors in the regression equation [5].

There is no consensus in the LUR community about the VIF cutoff that should be used for LUR model building. Henderson et al. (2007) [7], Clougherty et al. (2013) [19], Eeftens et al. (2012) [9], and Gulliver et al. (2013) [14] arbitrarily applied VIFs of ~1.5, 2, 3 and 3, respectively. However, O'brien (2007) suggests caution against removing potentially important variables from regression models with a stringent VIF [5], and Kutner et al. (2004) suggest a value of 10 as the rule of thumb for avoiding multicollinearity [18]. Although we chose this value for our analyses, the VIF of predictor variables was less than 4 in all cases.

The rationale for step 9

A recent analysis from Girona (Spain) demonstrated that LUR models should be restricted to a set of potential predictor variables [20]. We therefore decided to restrict the number of predictor variables in a LUR model to square root of number of measurement sites. We believe this restriction could provide more realistic R^2 values.

The rationale for step 10

This step is one of the assumptions of any regression analysis. In fact, the residuals of final regression model should be normally distributed [21] in order to have valid *p*-values for predictors' coefficients in regression model.

Table S2. The Spearman correlation coefficients between the annual (A), cooler season (C), and warmer season (W) measured concentrations at 23 fixed-sites for NO, NO_2, and NOx in 2010 in Tehran, Iran.

	A_NO	C_NO	W_NO	A_NO_2	C_NO_2	W_NO_2	A_NO_X	C_NO_X	W_NO_X
A_NO	1.00	-	-	-	-	-	-	-	-
C_NO	0.99	1.00	-	-	-	-	-	-	-
W_NO	0.96	0.94	1.00	-	-	-	-	-	-
A_NO_2	0.30	0.31	0.29	1.00	-	-	-	-	-
C_NO_2	0.25	0.25	0.27	0.92	1.00	-	-	-	-
W_NO_2	0.46	0.45	0.43	0.78	0.61	1.00	-	-	-
A_NO_X	0.94	0.94	0.92	0.54	0.50	0.63	1.00	-	-
C_NO_X	0.94	0.95	0.91	0.52	0.50	0.59	0.99	1.00	-
W_NO_X	0.87	0.85	0.89	0.47	0.37	0.71	0.92	0.90	1.00

Table S3. Final model results for annual NO. Variables are ordered by partial R^2, and those in bold indicate consistencies between models for the same pollutant.

R^2 = 0.78, Adjusted R^2 = 0.71, LOOCV R^2 = 0.66, *p*-value of the regression model = <0.001, RMSE = 32.1, Measured response, mean (min–max)= 88 (23–312), Moran's I = –0.08						
Predictors	Coefficients	SE	t	Partial R^2	*p*-value	VIF
Intercept	1.526e+00	6.930e-01	2.2	-	0.042	-
**DIST to TACZ**	–1.369e-04	2.524e-05	–5.4	0.63	<0.001	1.3
**LNDIST to PRSC**	6.942e-01	1.319e-01	5.3	0.62	<0.001	**1.8**
**GRS.500**	–3.103e-06	7.036e-07	–4.4	0.53	<0.001	1.1
**SLP**	–4.426e-02	1.059e-02	–4.2	0.51	<0.001	1.7
URF.100	–3.033e-05	1.379e-05	–2.2	0.22	0.042	1.1
For variables of the form XXX.YYY the XXX indicates the variable type, and the YYY indicates the buffer size, in meters.<br>Response of the model is Ln (annual NO); hence, predicted pollutant is Exp (model)						
Radius variable types included in the models were:<br>GRS = green space area<br>URF = urban facilities area						
The linear distance variable included in the model was:<br>DIST to TACZ = distance to traffic access control zone						
The log-linear distance variable included in the model was:<br>LNDIST to PRSC = log distance to the nearest primary school						
Other variable included in the model was:<br>SLP = slope						
Abbreviations: LOOCV, leave-one-out cross-validation; RMSE, root mean square error; SE, standard error; VIF, variance inflation factor						

Table S4. Final model results for cooler season NO. Variables are ordered by partial R^2, and those in bold indicate consistencies between models for the same pollutant.

<table>
<tr><td colspan="7">R^2 = 0.69, Adjusted R^2 = 0.60, LOOCV R^2 = 0.53, *p*-value of the regression model = <0.001, RMSE = 38.6, Measured response, mean (min–max)= 117 (30–358), Moran’s I = –0.09</td></tr>
<tr><td>Predictors</td><td>Coefficients</td><td>SE</td><td>t</td><td>Partial R^2</td><td>p-value</td><td>VIF</td></tr>
<tr><td>Intercept</td><td>1.919e+00</td><td>9.274e-01</td><td>2.2</td><td>-</td><td>0.054</td><td>-</td></tr>
<tr><td>LNDIST to PRSC</td><td>5.088e-01</td><td>1.412e-01</td><td>3.6</td><td>0.43</td><td>0.002</td><td>1.4</td></tr>
<tr><td>TPDC.2500</td><td>4.691e-05</td><td>1.822e-05</td><td>2.6</td><td>0.28</td><td>0.020</td><td>1.5</td></tr>
<tr><td>DIST to BST</td><td>–1.096e-04</td><td>4.266e-05</td><td>–2.6</td><td>0.28</td><td>0.020</td><td>1.2</td></tr>
<tr><td>DIST to PST</td><td>–3.205e-04</td><td>1.278e-04</td><td>–2.5</td><td>0.27</td><td>0.023</td><td>1.3</td></tr>
<tr><td>GRS.400</td><td>–2.088e-06</td><td>1.091e-06</td><td>–1.9</td><td>0.18</td><td>0.073</td><td>1.0</td></tr>
<tr><td colspan="7">For variables of the form XXX.YYY the XXX indicates the variable type, and the YYY indicates the buffer size, in meters.
Response of the model is Ln (cooler season NO); hence, predicted pollutant is Exp (model)</td></tr>
<tr><td colspan="7">Radius variable type included in the model was:
GRS = green space area</td></tr>
<tr><td colspan="7">The linear distance variables included in the model were:
DIST to BST = distance to bus terminal
DIST to PST = distance to petrol stations</td></tr>
<tr><td colspan="7">The log-linear distance variable included in the model was:
LNDIST to PRSC = log distance to the nearest primary school</td></tr>
<tr><td colspan="7">Other variable included in the model was:
TPDC = population density excluding unemployed and children <5 years</td></tr>
<tr><td colspan="7">Abbreviations: LOOCV, leave-one-out cross-validation; RMSE, root mean square error; SE, standard error; VIF, variance inflation factor</td></tr>
</table>

Table S5. Final model results for warmer season NO. Variables are ordered by partial R^2, and those in bold indicate consistencies between models for the same pollutant.

R^2 = 0.72, Adjusted R^2 = 0.64, LOOCV R^2 = 0.59, *p*-value of the regression model = <0.001, RMSE = 36.9, Measured response, mean (min–max)= 60 (17–268), Moran's I = –0.07						
Predictors	Coefficients	SE	t	Partial R^2	*p*-value	VIF
Intercept	6.793e-01	8.270e-01	0.82	-	0.423	-
**DIST to TACZ**	–1.551e-04	3.157e-05	–4.9	0.59	<0.001	1.5
**LNDIST to PRSC**	7.442e-01	1.571e-01	4.7	0.57	<0.001	1.8
**SLP**	–6.259e-02	1.440e-02	–4.3	0.53	<0.001	**2.2**
**GRS.500**	–2.021e-06	8.209e-07	–2.5	0.26	0.025	1.7
DIST to GRS	9.359e-04	3.992e-04	2.3	0.24	0.031	1.9
For variables of the form XXX.YYY the XXX indicates the variable type, and the YYY indicates the buffer size, in meters.<br>Response of the model is Ln (warmer season NO); hence, predicted pollutant is Exp (model)						
Radius variable type included in the model was:<br>GRS = green space area						
The linear distance variables included in the model were:<br>DIST to TACZ = distance to traffic access control zone<br>DIST to GRS = distance to green space area						
The log-linear distance variable included in the model was:<br>LNDIST to PRSC = log distance to the nearest primary school						
Other variable included in the model was:<br>SLP = slope						
Abbreviations: LOOCV, leave-one-out cross-validation; RMSE, root mean square error; SE, standard error; VIF, variance inflation factor						

Table S6. Final model results for annual NO_2. Variables are ordered by partial R^2, and those in bold indicate consistencies between models for the same pollutant.

$R^2 = 0.69$, Adjusted $R^2 = 0.62$, LOOCV $R^2 = 0.57$, *p*-value of the regression model = <0.001, RMSE = 9.9, Measured response, mean (min–max)= 53 (22–96), Moran's I = –0.07						
Predictors	Coefficients	SE	t	Partial R^2	*p*-value	VIF
Intercept	2.897e+00	3.813e-01	7.6	-	<0.001	-
**OFIC.300**	1.111e-05	2.295e-06	4.8	0.57	<0.001	1.2
**DIST to SNS**	–1.537e-04	3.984e-05	–3.9	0.45	0.001	1.0
**LNDIST to PRSC**	1.702e-01	6.211e-02	2.7	0.29	0.013	**1.3**
**OTHR.300**	2.214e-05	9.434e-06	2.3	0.23	0.031	1.2
For variables of the form XXX.YYY the XXX indicates the variable type, and the YYY indicates the buffer size, in meters.<br>Response of the model is Ln (annual NO); hence, predicted pollutant is Exp (model)						
Radius variable types included in the models were:<br>OFIC = official/commercial land use area<br>OTHR = other land use area						
The linear distance variable included in the model was:<br>DIST to SNS = distance to sensitive area						
The log-linear distance variable included in the model was:<br>LNDIST to PRSC = log distance to the nearest primary school						
Abbreviations: LOOCV, leave-one-out cross-validation; RMSE, root mean square error; SE, standard error; VIF, variance inflation factor						

Table S7. Final model results for cooler season NO_2. Variables are ordered by partial R^2, and those in bold indicate consistencies between models for the same pollutant.

R^2 = 0.75, Adjusted R^2 = 0.68, LOOCV R^2 = 0.58, p-value of the regression model = <0.001, RMSE = 9.2, Measured response, mean (min–max)= 62 (21–103), Moran's I = –0.03						
Predictors	Coefficients	SE	t	Partial R^2	*p*-value	VIF
Intercept	–5.711e+01	2.575e+01	–2.2	-	0.040	-
**OFIC.300**	5.946e-04	1.079e-04	5.5	0.64	<0.001	1.1
ELEV	1.062e-01	2.317e-02	4.6	0.55	<0.001	3.6
DIST to AIR	–4.120e-03	1.184e-03	–3.5	0.42	0.003	**3.8**
**OTHR.400**	8.346e-04	3.109e-04	2.7	0.3	0.016	1.3
ARD.100	–1.549e-03	6.866e-04	–2.3	0.23	0.038	1.1
For variables of the form XXX.YYY the XXX indicates the variable type, and the YYY indicates the buffer size, in meters.<br>Response of the model is (Ln (cooler season NO_2))^3; hence, predicted pollutant is Exp (model$^{0.33}$)						
Radius variable types included in the model were:<br>OFIC = official/commercial land use area<br>OTHR = other land use area<br>ARD = arid/undeveloped area						
The linear distance variable included in the model was:<br>DIST to AIR = distance to airport or air cargo facilities						
Other variable included in the model was:<br>ELEV = elevation						
Abbreviations: LOOCV, leave-one-out cross-validation; RMSE, root mean square error; SE, standard error; VIF, variance inflation factor						

Table S8. Final model results for warmer season NO_2. Variables are ordered by partial R^2, and those in bold indicate consistencies between models for the same pollutant.

R^2 = 0.64, Adjusted R^2 = 0.58, LOOCV R^2 = 0.51, *p*-value of the regression model = <0.001, RMSE = 10.2, Measured response, mean (min–max)= 45 (23–89), Moran's I = –0.11						
Predictors	Coefficients	SE	t	Partial R^2	*p*-value	VIF
Intercept	3.270e-01	2.551e-02	12.8	-	<0.001	-
**OFIC.300**	–6.812e-07	1.615e-07	–4.2	0.48	<0.001	1.1
**DIST to SNS**	1.179e-05	2.910e-06	4.1	0.46	<0.001	1.0
**LNDIST to PRSC**	–1.044e-02	4.259e-03	–2.5	0.24	0.024	**1.2**
For variables of the form XXX.YYY the XXX indicates the variable type, and the YYY indicates the buffer size, in meters.<br>Response of the model is (Ln (warmer season NO_2))$^{-1}$; hence, predicted pollutant is Exp (model^{-1})						
Radius variable type included in the model was:<br>OFIC = official/commercial land use area						
The linear distance variable included in the model was:<br>DIST to SNS = distance to sensitive area						
The log-linear distance variable included in the model was:<br>LNDIST to PRSC = log distance to the nearest primary school						
Abbreviations: LOOCV, leave-one-out cross-validation; RMSE, root mean square error; SE, standard error; VIF, variance inflation factor						

Table S9. Final model results for annual NO_X. Variables are ordered by partial R^2, and those in bold indicate consistencies between models for the same pollutant.

R^2 = 0.71, Adjusted R^2 = 0.62, LOOCV R^2 = 0.58, *p*-value of the regression model = <0.001, RMSE = 52.7, Measured response, mean (min–max)= 142 (66–385), Moran's I = –0.04						
Predictors	Coefficients	SE	t	Partial R^2	*p*-value	VIF
Intercept	1.526e+00	6.930e-01	7.8	-	<0.001	-
**DIST to TACZ**	2.017e-06	3.735e-07	5.4	0.63	<0.001	1.7
**LNDIST to PRSC**	–6.481e-03	1.679e-03	–3.9	0.47	0.001	1.7
DIST to OFIC	2.620e-05	7.121e-06	3.7	0.44	0.002	**1.7**
**LNDIST to HZRFAC**	–3.210e-03	1.097e-03	–2.9	0.33	0.009	1.4
**BGD.400**	–1.248e-03	5.629e-04	–2.2	0.22	0.041	1.3
For variables of the form XXX.YYY the XXX indicates the variable type, and the YYY indicates the buffer size, in meters.<br>Response of the model is (Ln (annual NO_X))$^{-2}$; hence, predicted pollutant is Exp (model$^{-0.5}$)						
The linear distance variables included in the model were:<br>DIST to TACZ = distance to traffic access control zone<br>DIST to OFIC = distance to official/commercial area						
The log-linear distance variables included in the model were:<br>LNDIST to PRSC = log distance to the nearest primary school<br>LNDIST to HZRFAC = log distance to hazardous facilities						
Other variable included in the model was:<br>BGD = product of bridge length in a buffer radii divide to distance to the bridges						
Abbreviations: LOOCV, leave-one-out cross-validation; RMSE, root mean square error; SE, standard error; VIF, variance inflation factor						

Table S10. Final model results for cooler season NO_X. Variables are ordered by partial R^2, and those in bold indicate consistencies between models for the same pollutant.

R^2 = 0.79, Adjusted R^2 = 0.73, LOOCV R^2 = 0.63, *p*-value of the regression model = <0.001, RMSE = 37.1, Measured response, mean (min–max)= 180 (76–435), Moran's I = –0.03						
Predictors	Coefficients	SE	t	Partial R^2	*p*-value	VIF
Intercept	2.941e-01	2.188e-02	13.4	-	<0.001	-
**DIST to TACZ**	5.800e-06	7.633e-07	7.6	0.77	<0.001	**1.9**
**LNDIST to HZRFAC**	–1.129e-02	2.462e-03	–4.6	0.55	<0.001	1.9
URF.100	1.648e-06	4.142e-07	4.0	0.48	<0.001	1.5
GRS.400	8.761e-08	2.322e-08	3.8	0.46	0.002	1.3
**LNDIST to PRSC**	–9.239e-03	2.780e-03	–3.3	0.39	0.004	1.1
For variables of the form XXX.YYY the XXX indicates the variable type, and the YYY indicates the buffer size, in meters.<br>Response of the model is (Ln (cooler season NO_X))$^{-1}$; hence, predicted pollutant is Exp (model^{-1})						
Radius variable types included in the model were:<br>GRS = green space area<br>URF = urban facilities area						
The linear distance variable included in the model was:<br>DIST to TACZ = distance to traffic access control zone						
The log-linear distance variables included in the model were:<br>LNDIST to HZRFAC = log distance to hazardous facilities<br>LNDIST to PRSC = log distance to the nearest primary school						
Abbreviations: LOOCV, leave-one-out cross-validation; RMSE, root mean square error; SE, standard error; VIF, variance inflation factor						

Table S11. Final model results for warmer season NO_X. Variables are ordered by partial R^2, and those in bold indicate consistencies between models for the same pollutant.

R^2 = 0.61, Adjusted R^2 = 0.50, LOOCV R^2 = 0.42, *p*-value of the regression model = 0.004, RMSE = 44.8, Measured response, mean (min–max)= 105 (49–336), Moran's I = –0.08						
Predictors	Coefficients	SE	t	Partial R^2	*p*-value	VIF
Intercept	7.106e-03	1.400e-03	5.1	-	<0.001	-
**LNDIST to PRSC**	–8.153e-04	2.373e-04	–3.4	0.41	0.003	**1.9**
**DIST to TACZ**	1.286e-07	4.798e-08	2.7	0.3	0.016	1.5
$(OFIC.100)^{0.1}$	–3.977e-04	1.531e-04	–2.6	0.28	0.019	1.6
**BGD.400**	–1.349e-04	7.224e-05	–1.9	0.17	0.079	1.2
SLP	3.376e-05	1.813e-05	1.9	0.17	0.080	1.6
For variables of the form XXX.YYY the XXX indicates the variable type, and the YYY indicates the buffer size, in meters.<br>Response of the model is (Ln (warmer season NO_X))$^{-4}$; hence, predicted pollutant is Exp (model$^{-0.25}$)						
Radius variable types included in the models were:<br>OFIC = official/commercial land use area						
The linear distance variable included in the model was:<br>DIST to TACZ = distance to traffic access control zone						
The log-linear distance variable included in the models was:<br>LNDIST to PRSC = log distance to the nearest primary school						
Other variable included in the model was:<br>BGD = product of bridge length in a buffer radii divide to distance to the bridges<br>SLP = slope						
Abbreviations: LOOCV, leave-one-out cross-validation; RMSE, root mean square error; SE, standard error; VIF, variance inflation factor						

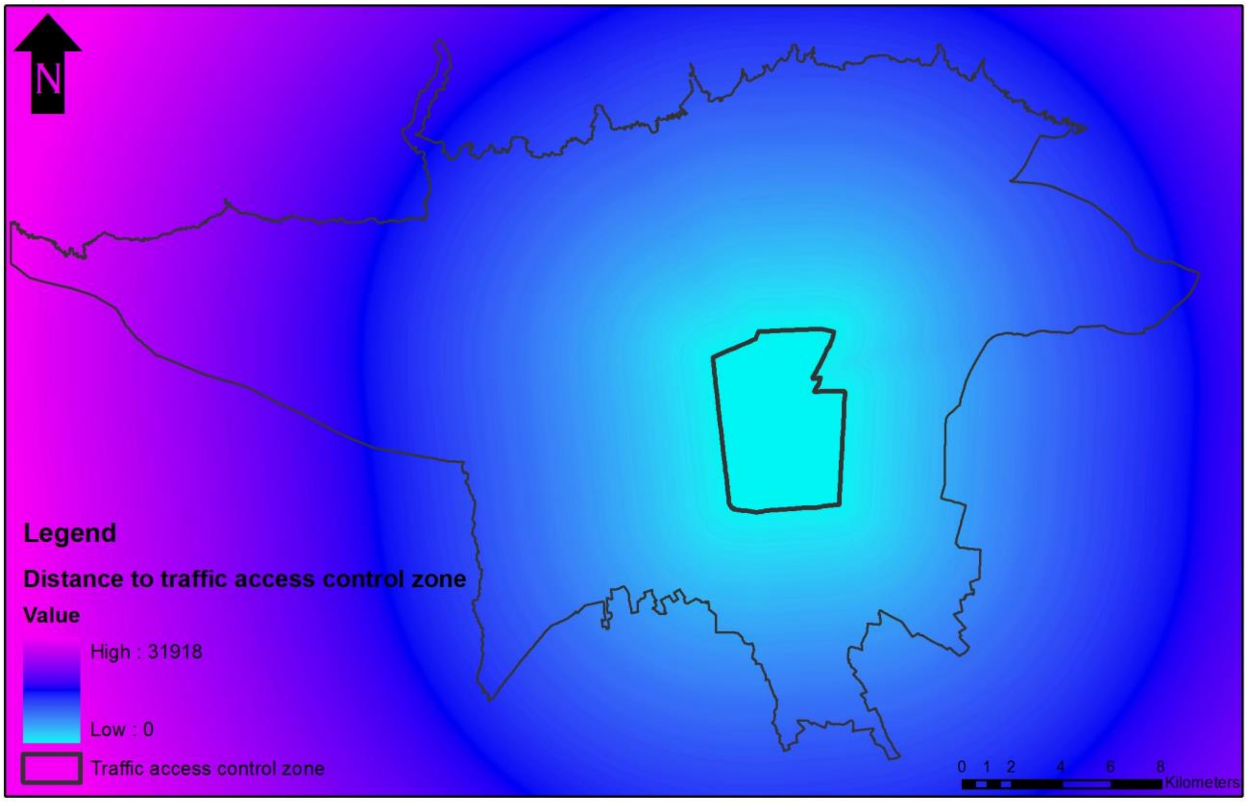

Figure S3. Spatial variability of distance to traffic access control zone (TACZ) in 2010 in Tehran, Iran. The figure is generated using ESRI's ArcGIS 10.2.1 for Desktop (http://www.esri.com/) [22].

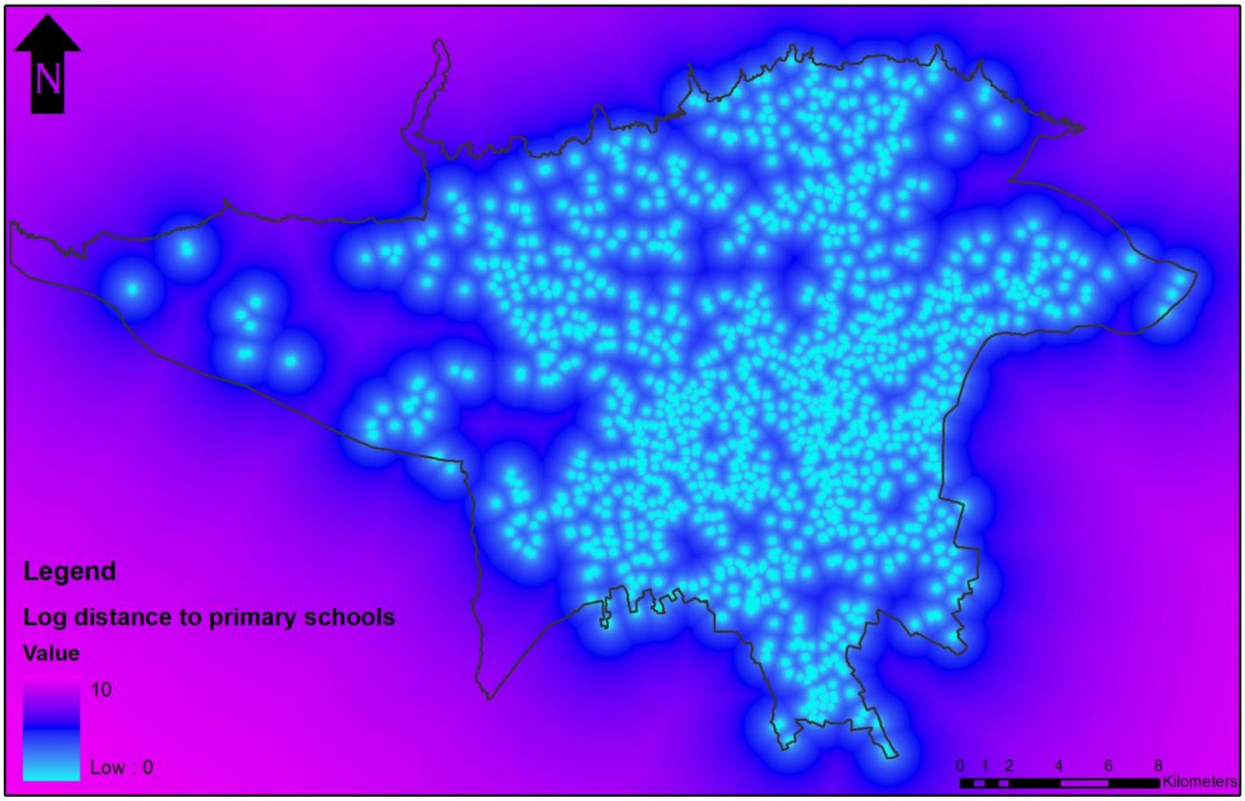

Figure S4. Spatial distribution of primary schools in Tehran (in 2010) and natural logarithm of distance to the nearest primary school. The figure is generated using ESRI's ArcGIS 10.2.1 for Desktop (http://www.esri.com/) [22].

References

1 Honaker, J., King, G. & Blackwell, M. Amelia II: A program for missing data. *J. Stat. Softw.* **45**, 1-47 (2011).

2 Clayton, D., Hills, M. & Pickles, A. *Statistical models in epidemiology*. Vol. 161 (IEA, 1993).

3 Amini, H. *et al.* Land use regression models to estimate the annual and seasonal spatial variability of sulfur dioxide and particulate matter in Tehran, Iran. *Sci. Total Environ.* **488**, 343-353 (2014).

4 Efron, B. & Gong, G. A leisurely look at the bootstrap, the jackknife, and cross-validation. *Am. Stat.* **37**, 36-48 (1983).

5 O'brien, R. M. A caution regarding rules of thumb for variance inflation factors. *Qual. Quant.* **41**, 673-690 (2007).

6 Jarque, C. M. & Bera, A. K. Efficient tests for normality, homoscedasticity and serial independence of regression residuals. *Economics letters* **6**, 255-259 (1980).

7 Henderson, S. B., Beckerman, B., Jerrett, M. & Brauer, M. Application of land use regression to estimate long-term concentrations of traffic-related nitrogen oxides and fine particulate matter. *Environ. Sci. Technol.* **41**, 2422-2428 (2007).

8 Hoek, G. *et al.* Land use regression model for ultrafine particles in Amsterdam. *Environ. Sci. Technol.* **45**, 622-628 (2010).

9 Eeftens, M. *et al.* Development of land use regression models for PM2.5, PM2.5 absorbance, PM10 and PM coarse in 20 European study areas; Results of the ESCAPE project. *Environ. Sci. Technol.* **46**, 11195-11205, doi:10.1021/es301948k (2012).

10 Gonzales, M. *et al.* Evaluation of land use regression models for NO2 in El Paso, Texas, USA. *Sci. Total Environ.* **432**, 135-142 (2012).

11 Beelen, R. *et al.* Development of NO2 and NOx land use regression models for estimating air pollution exposure in 36 study areas in Europe–the ESCAPE project. *Atmos. Environ.* (2013).

12 de Hoogh, K. *et al.* Development of land use regression models for particle composition in 20 study areas in Europe. *Environ. Sci. Technol.*, doi:10.1021/es400156t (2013).

13 Abernethy, R. C., Allen, R. W., McKendry, I. G. & Brauer, M. A land use regression model for ultrafine particles in Vancouver, Canada. *Environ. Sci. Technol.* (2013).

14 Gulliver, J., de Hoogh, K., Hansell, A. & Vienneau, D. Development and back-extrapolation of NO2 land use regression models for historic exposure assessment in Great Britain. *Environ. Sci. Technol.* (2013).

15 Gilbert, N. L., Goldberg, M. S., Beckerman, B., Brook, J. R. & Jerrett, M. Assessing spatial variability of ambient nitrogen dioxide in Montreal, Canada, with a land-use regression model. *J. Air Waste Manage. Assoc.* **55**, 1059-1063 (2005).

16 Ryan, P. H. *et al.* A land-use regression model for estimating microenvironmental diesel exposure given multiple addresses from birth through childhood. *Sci. Total Environ.* **404**, 139-147 (2008).

17 Basagaña, X. *et al.* Effect of the number of measurement sites on land use regression models in estimating local air pollution. *Atmos. Environ.* **54**, 634-642 (2012).

18 Kutner, M. H., Nachtsheim, C. & Neter, J. *Applied linear regression models*. (McGraw-Hill, 2004).

19 Clougherty, J. E. *et al.* Intra-urban spatial variability in wintertime street-level concentrations of multiple combustion-related air pollutants: The New York City Community Air Survey (NYCCAS). *J. Expo. Sci. Environ. Epidemiol.* (2013).
20 Basagaña, X. *et al.* Effect of the number of measurement sites on land use regression models in estimating local air pollution. *Atmospheric Environment* **54**, 634-642 (2012).
21 Cook, R. D. & Weisberg, S. Residuals and influence in regression. (1982).
22 ESRI 2016. ArcGIS Desktop: Release 10.2.1. Redlands, CA: Environmental Systems Research Institute (http://www.esri.com/).
